# Posterior Drainage in Gunshot Wounds of the Abdomen

**Published:** 1902-08-16

**Authors:** 


### POSTERIOR DRAINAGE IN GUNSHOT WOUNDS OF THE ABDOMEN.

In the course of the South African war we have heard of many extraordinary cases of recovery after perforation of the abdomen by bullet wounds. Seldom, however, has so good a case occurred as one which was related by Dr. Roswell Park [1] to the recent meeting of the American Surgical Association. In this case the wound was made by a revolver bullet in the middle line, just an inch above the tip of the xyphoid. This bone was shattered, and when by a double incision the whole area was incised the abdominal cavity was found full of clots and detritus. With his hands he scooped out more than two quarts of this stuff, and then was able to make an examination of the parts. The left lobe of the liver was deeply cut; the stomach was perforated, but fortunately had been comparatively empty; it was therefore not washed, and the wounds were closed by triple sutures. No search was made for the bullet, it having been shown that such efforts are worse than useless. Posterior drainage was then established through an incision just below the tenth rib, and a tube was passed into the lesser peritoneal cavity. Recovery was uninterrupted and complete. In the discussion on the subject, although a good deal of support was given to the practice of posterior drainage, there was by no means unanimity on the subject, and among the objections raised was one which was probably not entirely inapt, namely, that posterior drainage might occasionally open the pleural cavity. A point made by one of the speakers is worth referring to, namely, the importance of wetting a gauze drain thoroughly before it is inserted. Both the gauze drain and the first compress, it was said, should be thoroughly saturated before being applied if they were to draw the serum properly from the depths of a wound. If this is true it is a matter of some moment, for it certainly is not always done.

[1] New York Medical News, July 26.

